# Supplementary material for: Reservoir-computing based associative memory and itinerancy for complex dynamical attractors
Source: Nat Commun. 2024 Jun 6;15:4840. doi: 10.1038/s41467-024-49190-4 (PMC11156990; doi:10.1038/s41467-024-49190-4)
Supplement: Supplementary file 1 — Supplementary Information [file 41467_2024_49190_MOESM1_ESM.pdf]

Supplementary Information for

Reservoir-computing based associative memory and itinerancy for complex dynamical attractors

Ling-Wei Kong, Gene A. Brewer, and Ying-Cheng Lai

Corresponding author: Ying-Cheng Lai (Ying-Cheng.Lai@asu.edu)

CONTENTS

Supplementary Note 1. Reservoir Computing 2

Supplementary Note 2. Details of the dynamical attractors to be memorized 5

Supplementary Note 3. Fidelity of recalled attractors in the long term 6

Supplementary Note 4. Performance of index-based reservoir memory for memorizing 16 chaotic attractors 7

Supplementary Note 5. Reservoir-computing based attractor classifier 7

Supplementary Note 6. Effects of index values on the functional regions of artificial neurons in the reservoir network 8

Supplementary Note 7. Basin structures in index-based reservoir memory and switching success rates 8

Supplementary Note 8. Performance of our feedback control strategy for higher switch success rates under different parameters 9

Supplementary Note 9. Dynamical process of retrieval in index-free reservoir-computing memory 10

Supplementary Note 10. Dependency of memory retrieval in index-free reservoir computers with partial information on the specific missing dimensions 10

Supplementary Note 11. Effects of noise and random itinerancy 11

Supplementary figures 11

Supplementary References 28

References 28

Supplementary Note 1. RESERVOIR COMPUTING

We employ two types of RC networks for associative memory of complex dynamical attractors: index-based and index-free. Their architectures are not identical, but their training and testing methods (for storing and retrieving attractors) bear only minor differences. Therefore, in the following, we will describe the training and testing methods of both index-based and index-free schemes together. The codes for training and predicting with both architectures can be found at Ref. [1].

As shown in Fig. 1 in the main text, a reservoir computer consists of three layers: an input layer, a hidden recurrent layer, and an output layer. The major advantage of reservoir computing compared with most RNN architectures is that not all the weights in the layers need to be trained, and the training usually does not require backpropagation. Rather, a regularized linear regression suffices to train only the weights W_{out} of the output layer. This training scheme not only significantly lowers the training computational cost, but also helps mitigate issues such as catastrophic forgetting and vanishing/exploding gradient. These features make reservoir computing particularly suitable for our multifunctional tasks, which require training with a large set of training data sequences with distinct behaviors without forgetting.

The training of a reservoir neural network for memorizing dynamical attractors, whether index-based or index-free, can be divided into three steps. In the first step, we generate the input and recurrent hidden layers. They are generated with random values for the entries and fixed once generated. Specifically, in an index-based RC, there are two input matrices: the state input matrix W_{u} that projects the low-dimensional state vector $\mathbf{u}$ characterizing the target attractor to be memorized to the hidden layer, and the index input matrix W_{index} that injects the index value p associated with each attractor to be memorized into the reservoir neural network. The entries of both W_{u} and W_{index} are randomly generated by a uniformed distribution in the interval $[-c_{\text{in}}, c_{\text{in}}]$. Here c_{in} is a hyperparameter to be optimized. When we input the index value $p(t)$, since the specific values of p can be chosen rather arbitrarily, we introduce a linear transformation of it $k_p(p(t) + b_p)$ before injecting to the RC network to make sure it is not way too large or way too small. Here k_p and b_p are two hyperparameters. For index-free RCs, we only have W_{u} in the input layer, and there is no $p(t)$, W_{index}, k_p, or b_p. The RNN in the hidden layer has N neurons connected with each other by a network called "the reservoir". This reservoir network can be represented by the matrix W_{r}, with N as the network size. A basic parameter characterizing the network connectivity is the probability that a random pair of nodes is connected. We write this connectivity coefficient as s_r, which is another hyperparameter. The reservoir network is usually a sparse network with a small s_r, as in all the RCs used in this study. This sparsity significantly reduces the computational costs in both training and testing. The reservoir network is directed and weighted, where all the connecting weights are initially independently generated by a uniform distribution in the interval of $[0, 1]$. The entire matrix W_{r} is then rescaled so that the network spectral radius ρ (another hyperparameter) equals the desired value we obtain through the hyperparameter optimization.

In the second step, which is often called the "echoing step", the time series of each attractor is the input vector signal $\mathbf{u}(t)$ into the input layer and generates a response vector signal $\mathbf{r}(t)$ of equal length in the hidden layer. We call this process as the echoing process as we are simply observing the echoing of the input training signals in hidden layer (the reservoir). For the index-based memory, the corresponding index value $p(t)$ (a scalar piecewise constant function) is simultaneously

injected into the hidden-layer network. The equation of iteration in this echoing step is as follows:

$$\mathbf{r}(t) = (1-\alpha)\mathbf{r}(t-\Delta t) + \alpha \tanh\{W_{\text{r}} \cdot \mathbf{r}(t-\Delta t) \\ + W_{\text{u}} \cdot [\mathbf{u}(t) + \sigma_{\text{train}}\xi(t)] + W_{\text{index}} k_p [p(t) + b_p]\}, \tag{S1}$$

where α is the leakage parameter, and Δt is the time step of network dynamical evolution. For index-free memory retrieval, the term $W_{\text{index}} k_p [p(t) + b_p]$ is absent. Training noise is applied to better stabilize the memory states [2], and we have $\xi(t)$ term in the iteration equation as a standard Gaussian white noise added to the input data. The hyperparameter σ_{train} controls the magnitude of this noise. Since we are training with multiple target states, we do the echoing process for each of them sequentially. At the beginning of the echoing process for each target state, $\mathbf{r}(t)$ is initiated with all zeros. We then have a 10-step washing-out period immediately after the initialization to exclude the transient behaviors. After this washing-out period, all the hidden state $\mathbf{r}(t)$ are recorded to be used later in the second step. At the end of this first step (echoing step), we should have gathered a huge collection of hidden state $\mathbf{r}(t)$ with a number of the number of target states multiplied by the subtraction of the training length by washing out length.

In the third step, a regularized linear regression is carried out between the target state and the reservoir hidden state $\mathbf{r}(t)$ we have collected from the first step. In our cases, as we want the RC network to learn the dynamic rules of the target states, the training target is the same as the training inputs but with one time step forward. The RC network is thus essentially trained to make a one-step-ahead prediction of the target state.

Compared with a more standard approach where only one attractor/state is trained, the trick with our approach (for both indexed and index-free schemes) is that we then concatenate the records of the hidden states $\mathbf{r}(t)$ from different target states together in the temporal dimension to form one (potentially very long) time series $R(t)$. The training target is processed in the same way, where $\mathbf{v}(t)$ from different states are concatenated in the temporal dimension to form one (also potentially very long) time series $V(t)$. This "concatenating in time" scheme has been used in several previous work in the context of reservoir computing [3–5] when more than one state/attractor is trained. It is pretty interesting that this simple method works at all, especially given that the different states it concatenates can have very different dynamical features, from simple periodic oscillations to various chaotic trajectories with different forms of nonlinearity. The high dimensionality of the RC system and the simplicity of linear regression allow this way of merging different dynamics into one RNN. This is particularly helpful for our tasks with multiple states to memorize as no forgetting issue would arise.

One final treatment before the actual linear regression is that we follow the trick from Ref. [6, 7] to take a square of the even rows/neurons of $R(t)$ to form $R'(t)$ to exclude undesired potential symmetries in the RC system. Then, finally, a ridge regression is performed between $V(t)$ and $R(t)$, by the following equation:

$$W_{\text{out}} = V \cdot R'^T (R' \cdot R'^T + \beta I)^{-1}, \tag{S2}$$

where β is the l-2 regularization coefficient, another hyperparameter of the reservoir computer, and I is an identity matrix. Now we have our readout matrix from the reservoir W_{out}, and the training is finished.

A network so trained can serve as a closed-loop dynamical system capable of generating arbitrarily long trajectories of a memorized attractor after a successful recall according to the following

iterative dynamical equations:

$$
\begin{aligned}
\mathbf{r}(t) &= (1-\alpha)\mathbf{r}(t-\Delta t) + \alpha \tanh[W_{\text{r}} \cdot \mathbf{r}(t-\Delta t) \\
&\qquad + W_{\text{u}} \cdot \mathbf{u}(t) + W_{\text{index}} k_p (p(t) + b_p)], && \text{(S3)} \\
\mathbf{v}(t) &= W_{\text{out}} \cdot \mathbf{r}'(t), && \text{(S4)} \\
\mathbf{v}(t) &\to \mathbf{u}(t+\Delta t), && \text{(S5)}
\end{aligned}
$$

where $\mathbf{v}(t)$ is the output of the reservoir computer and should be the target state we want if the retrieval is successful. In the loop of generating a trajectory, the output $\mathbf{v}(t)$ becomes the state input $\mathbf{u}(t+\Delta t)$ of the next time step so that the iteration can continue for an arbitrary length. (This does not necessarily mean this arbitrarily long prediction is always accurate.) Again, the term $W_{\text{index}} k_p(p(t) + b_p)$ will disappear if we are operating an index-free reservoir computer.

For reservoir computing, hyperparameter optimization is often necessary. The hyperparameters that need optimization are the leakage α, the regularization coefficient β of the linear regression in training, the scale of the input matrix c_{in}, the spectral radius ρ of W_{r}, and the reservoir network connectivity s_r, and the strength of training noise σ_{train}. We use a Bayesian optimization algorithm (*surrogateopt* in Matlab). In such an optimization process, we go through a loop of iterations. In each iteration, we test a set of hyperparameters' values and receive a validation result. The algorithm uses the results we collect from previous iterations to fit a performance landscape in the space of the hyperparameters and use such a fitting to guide our future search for optimal hyperparameters. After a maximum iteration number is reached, we stop the iteration and select the set of hyperparameters with the best validation performance that we have tested. Compared with this Bayesian approach, a random search is very inefficient as each iteration is independent and history information is not utilized. A grid search is also not applicable as we have many different hyperparameters, so the parameter space's dimensionality is too high.

Table S1 lists all the specific values of all the hyperparameters that we get from the optimization and use in this paper. With different tasks and different training approaches, we have multiple groups of reservoir computers. The reservoir computers in the same group use the same set of hyperparameters. To make things clear, we assign each group a name, which is shown in the first column of Tab. S1. The group Indexed #1 refers to the indexed memory RCs that are trained with the six different memory states shown in Fig. 1(B) in the main text and Fig. S1. Results that use this set are shown in Fig. 1(B), Figs. 2(A, B, C, D, E, F), and Fig. 3 in the main text. Results shown in Figs. S2, S7, S9, and S15 also use this set. The group Indexed #2 refers to the indexed memory RCs that are trained to have 16 Sprott chaotic states, as shown in Fig. 1(C) in the main text. Results that use this set are shown in Figs. 2(G, H, I) in the main text, Figs. S3, S4, and S8. For the results in Figs. 2(G, H, I) in the main text and Fig. S8, the training length is $T_{\text{train}} = 4,000$ steps for each attractor. and the network size N is $N = 2,000$. For the results in Figs. S3 and S4, the training length is $T_{\text{train}} = 5,000$ steps for each attractor, and the network size is $N = 3,000$. The group Indexed #3 refers to the indexed memory RCs that are used in generating some of the scaling laws. More specifically, it is used in the "one-hot coding" task, the "binary coding" task, the "separate W_{out}" task, and the "bifurcation task" in Fig. 4(A) in the main text. It is also used in all the curves in Figs. 4(B,C) in the main text, Figs. S10 and S11. The training length T_{train} is 1,000 steps for all these results except the "bifurcation" task where the training length T_{train} is 2,000 steps. The group Indexed #4 refers to the indexed memory RCs that are used in the "ALOI" task in Fig. 1(A) in the main text. The group Index-Free #1 refers to the index-free memory RCs that are trained with the

RC Group	N	T_{train}	ρ	c_{in}	α	$\log_{10}\beta$	s_r	$\log_{10}\sigma_{\text{train}}$	k_p	b_p
Indexed #1	1,000	6,000	0.78	0.85	0.37	-7.5	0.21	-3.1	1.12	-1.08
Indexed #2	2,000 or 3,000	4,000 or 5,000	0.39	0.91	0.64	-6.5	0.4	-3	3.3	-10
Indexed #3	-	1,000 or 2,000	0.39	0.91	0.64	-6.5	0.005	-3	-	-
Indexed #4	-	1,440	0.3	0.9	0.6	-6.5	0.005	-2	1	0
Index-Free #1	4,000	6,000	1.47	1.13	1	-6.4	0.19	-2.9	-	-
Index-Free #2	-	1,000	1.92	2.82	0.42	-7.1	0.0027	-4.5	-	-

TABLE S1. Hyperparameters of the reservoir computers used in this paper. The parts of results in the paper that used each RC group are discussed in the Methods section, with more details on the specific choices of training length T_{train} and reservoir size N. The training length T_{train} refers to the number of steps used in the training for each target state.

six different memory states shown in Fig. 1B) in the main text and Fig. S1. Results that use this set are shown in Figs. 5, 6, and 7 in the main text, as well as Figs. S12, S13, S14, and S16. The group Index-Free #1 refers to the index-free memory RCs used in the "index-free" task in Fig. 1(A) in the main text.

Supplementary Note 2. DETAILS OF THE DYNAMICAL ATTRACTORS TO BE MEMORIZED

We describe how the training and testing data for the target attractors to be memorized are generated. All the data used in work can be found under the 'data' folder in our GitHub repository [8].

The training data $\mathbf{u}(t) = [u_x(t), u_y(t), u_z(t)]^T$ for the six attractors in Fig. 1(B) in the main text (illustrated in Fig. S1), are generated as follows.

- A periodic Lissajous system with the frequency ratio as 1:3:5, where $\mathbf{u}(t)$ is generated by the following formulas with time step $\Delta t = 1$.

$$\begin{aligned} u_x(t) &= \sin(\pi t/100), \\ u_y(t) &= \sin(3\pi t/100 + \pi/2), \\ u_z(t) &= \sin(\pi t/20). \end{aligned} \tag{S6}$$

- A periodic attractor from the Sprott system [9] is generated by the following equations:

$$\begin{aligned} du_x/dt &= 0.3u_x + u_z, \\ du_y/dt &= u_x u_z - u_y, \\ du_z/dt &= -u_x + u_y. \end{aligned} \tag{S7}$$

The time step (temporal resolution) in the training data is $\Delta t = 0.015$.

- The classic chaotic Lorenz attractor is generated from the equations

$$\begin{aligned} du_x/dt &= 10(u_y - u_x), \\ du_y/dt &= u_x(28 - u_z) - u_y, \\ du_z/dt &= u_x u_y - 8/3 u_z. \end{aligned} \tag{S8}$$

The time step (temporal resolution) in the training data is $\Delta t = 0.02$.

- The classic chaotic Rössler system is generated from

$$
\begin{aligned}
du_x/dt &= -u_y - u_z, \\
du_y/dt &= u_x + 0.2u_y, \\
du_z/dt &= u_z(u_x - 5.7) + 0.2.
\end{aligned} \tag{S9}
$$

  The time step (temporal resolution) in the training data is $\Delta t = 0.1$.

- A chaotic attractor from the food chain system [10] is generated by the following equations:

$$
\begin{aligned}
du_x/dt &= u_x - \frac{0.2u_x u_y}{1 + 0.05u_x}, \\
du_y/dt &= -u_y + \frac{0.2u_x u_y}{1 + 0.05u_x} - u_y u_z, \\
du_z/dt &= -10(u_z - 0.006) + u_y u_z.
\end{aligned} \tag{S10}
$$

  The time step (temporal resolution) in the training data is $\Delta t = 0.15$.

- A chaotic attractor from the Hindmarsh-Rose (HR) neuron system [11] is generated from the following equations:

$$
\begin{aligned}
du_x/dt &= u_y - u_x^3 + 3u_x^2 - u_z + 3.25, \\
du_y/dt &= 1 - 5u_x^2 - u_y, \\
du_z/dt &= 0.006(4(u_x + 8/5) - u_z).
\end{aligned} \tag{S11}
$$

  The time step (temporal resolution) in the training data is $\Delta t = 0.6$.

Supplementary Note 3. FIDELITY OF RECALLED ATTRACTORS IN THE LONG TERM

In the main text, we discuss how we evaluate the maximum Lyapunov exponents and the correlation dimensions of the reconstructed chaotic attractors in the retrieval phase to validate the fidelity of the reconstructed attractor accuracy. Here we provide more results on this point, by calculating the maximum Lyapunov exponents of the reconstructed attractors under different levels of training noise σ_{train}. The result is shown in Fig. S2. We show that there is an optimal training noise region where the maximum Lyapunov exponents of most of the reconstructed chaotic attractors agree well with the ground truth values.

Note that this "fidelity of recalled attractors in the long term" is a separate issue from achieving "long-term memory", although both expressions have something to do with a long time scale. It is also indeed true, though, that our framework satisfies both "long-term" criteria. The former criterion requires that once a memory state is recalled, it can persist for a long term without losing or deviating from the crucial dynamical features of the target memory state (such as the maximum Lyapunov exponent). The latter criterion requires that the dynamical information is stored in the weights and connections of the Rc network, not the hidden state, so that the memory states can be recalled with proper cues or other recalling methods, even with a random initial hidden state. Thus, a recalled state that only persists in the memory device for several periods may still be considered as "long-term memory". In other words, the "term" in the name "long-term memory" actually refers to the time *between* memorizing and recalling, not to the time length *of* recalling.

Supplementary Note 4. PERFORMANCE OF INDEX-BASED RESERVOIR MEMORY FOR MEMORIZING 16 CHAOTIC ATTRACTORS

In Fig. 1(C) in the main text, the 16 chaotic attractors to be memorized and the 2D index values are displayed. Here we provide the results of testing (retrieval), as shown in Fig. S3, where the blue and red trajectories are the ground truth and the outputs of reservoir memory, respectively. The length of the recalled trajectories can be arbitrarily long.

In Fig. S4, we test if a randomized assignment of the index values will harm the performance. We show that, in most cases, the reservoir computer can successfully recall the target memory states and accurately persist the state for at least four average periods. This result suggests that our approach can work on this dataset regardless of how the index values are assigned.

Supplementary Note 5. RESERVOIR-COMPUTING BASED ATTRACTOR CLASSIFIER

We use a classifier reservoir computer to classify the outputs of the reservoir memory system in an automated way and to check if the output trajectory agrees with that of the desired attractor. In the main text, the classifier RC is used in two tasks. The first is in the application of random perturbation and feedback control strategy to enhance the switching success rate with the index-based reservoir memory. The classifier reservoir computer, as described in Figs. 3(D), is deployed inside the feedback loop to determine if the desired attractor has been reached and if further perturbation is required. The second task is with index-free reservoir computers, where a classifier is used to distinguish the retrieved trajectories and to produce the retrieval success rate. A classifier is necessary here as no explicit ground truth trajectory can be found for the chaotic target states, so we cannot use measurements such as RMSE or prediction horizon.

A reservoir-computing-based classifier has three layers: an input layer, a recurrent hidden layer, and an output layer, but without any index channel. The input signal is the three-dimensional time series from the training data or the output of the reservoir memory, where the latter is an m-dimensional one-hot vector $v_c(t)$ at each time step. Each dimension of the output of the classifier is associated with one memorized attractor. For a well-trained classifier, the ith entry of $v_c(t)$ being approximately one implies that the time series is classified as in the ith memorized attractor at this time t. On the contrary, if the ith entry of $v_c(t)$ is approximately zero, then the time series will be classified as not belonging to the ith memorized attractor at this time. More specifically, the hyperparameters of the classifier RC are shown in Tab. 1. The training is performed on each of the six target states ten individual times, each time with a training length of 500 steps. The training procedure is the same as training an index-free memory RC, except that the output target is changed to one-hot coding vectors.

After training, we apply the trained classifier RC to the output time series of the memory RCs that we collect from the two tasks discussed above. These output time series of the memory RCs become the inputs of the classifier RC. Some exemplary inputs and outputs of the classifier RC are shown in Fig. S5. If the time series is an accurate reconstruction of the target state, we should observe a dark stripe in the output of classifier RC at the correct row. A failed retrieval is shown as the second example in the fourth row where, after recalling, the correct Rössler chaotic attractor appeared for a short period of transient time before the reservoir memory switched to a different state. It can be seen that the stripe in the output at index $p = 4$ breaks, and a new but wrong stripe at index $p = 6$ is formed. Our criterion in distinguishing an accurate and stable reconstruction of

any of the target states versus a failed one (Either an untrained state is activated, or the retrieval is too unstable and does not stay in one state for the majority of the classifying window.) is as follows. We take the row of the classifier RC output that has the maximum mean value, and test if this mean value is within the interval $[0.75, 1.25]$ around the ideal value 1. We then also look at the mean values of other rows, to see if none of these mean values are larger than 0.25. If the answers to both of the tests are true, then the classifier has classified this recall as an accurate and stable one of the target states that corresponds to the row with the maximum mean value. The testing window is always set to 300 steps, with a 50-step washing-out period.

A confusion matrix is demonstrated in Fig. S6, with 1,200 trials collected from the outputs from 8 different index-free memory reservoir computers. The accuracy is very high, where only in 6 trials there is a disagreement between the classifier RC and the human labeler. All these 6 trials are caused by the boundary between successful recalls (of any target state) and failed recalls (none of the target states is accurately and stably reconstructed during the classifying window). This boundary is indeed hard to define. No confusion among the target states is found in all the 1,200 trials.

Supplementary Note 6. EFFECTS OF INDEX VALUES ON THE FUNCTIONAL REGIONS OF ARTIFICIAL NEURONS IN THE RESERVOIR NETWORK

It is shown in the main text that the index values modify the bias terms in the artificial neurons in the reservoir network, thus affecting the functional regions in the activation function. Here, we explicitly demonstrate this effect.

The attractors to be memorized are dynamical trajectories with periodic or chaotic oscillations. When an oscillatory signal is fed into the recurrent neural network in the hidden layer, most neurons will be excited to oscillate as well. For the index-based reservoir memory, the oscillatory patterns of the neurons can be tuned by the index values, as shown in Fig. S7. Corresponding to the case of memorizing six attractors with a one-dimensional index, we calculate the maximum/median/minimum values of the oscillatory pattern of each neuron for two different attractors, displayed in the left and right columns of Fig. S7, respectively. It can be seen that, for neurons with different p_i values, the maximum/median/minimum values of their oscillations are different, indicating that the oscillatory patterns of the neurons can indeed be tuned by the index value (through W_{index}). Remarkably, Figs. S7(B) and S7(E) show that $\tanh(W_{\text{index}} p_i)$ fits the median values of the oscillating neurons quite well. Overall, changing the index value can alter the oscillatory patterns in the reservoir network, making storage of independent attractors possible without the need for multistablity in the high-dimensional phase space of the dynamical network in the hidden layer.

Note that for the index-free reservoir memory system, because of the absence of index values, multistablity is necessary to realize any memory capacity. The occurrence of multistablity typically requires larger networks for the same task than index-based reservoir memory.

Supplementary Note 7. BASIN STRUCTURES IN INDEX-BASED RESERVOIR MEMORY AND SWITCHING SUCCESS RATES

Figure 2(F) in the main text shows that the variance among the average rates of each column $\text{Var}(\sum_i \eta_{i,j}/K)$ is much larger than those among the rows $\text{Var}(\sum_j \eta_{i,j}/K)$, where $\eta_{i,k}$ is

the success rate of switching from attractor s_i to attractor s_j. This indicates that the success rate of switching among memorized attractors depends more on the destination attractor than on the starting attractor.

To understand this dependence, we use the example Fig. 2(G) in the main text with 16 stored chaotic attractors. Choosing attractor $\#11$ as an example, we locate the regions in the 3D attractor generated by the reservoir memory where switching starts and distinguish those with successful (darker blue) and failed (orange) switchings, as shown in Fig. S8(A), where the ten panels correspond to ten different destination attractors (attractors Nos. 1, 2, 3, 4, 5, 6, 10, 13, 15, and 16, respectively). The success rate of switching can be estimated as the ratio between the numbers of blue and orange points. It can be seen that, on the starting attractor ($\#11$), the regions leading to successful switching to different destination attractors are different with distinct relative sizes and structures. For instance, the third panel in the first row gives a riddled structure, while the second panel in the second row has a smooth boundary between the blue and orange regions. These patterns are related to the basin structures of the destination attractors.

It is useful to further investigate the basin structures of the memorized attractors in the high-dimensional phase space of the reservoir network. The results are summarized in Fig. S8(B), where the thirty panels are organized as ten vertical columns, each corresponding to a panel (a distinct destination attractor) in Fig. S8(A), in the same order. In each column, the three panels show three 2D slices of the basin structure in the N-dimensional phase space, respectively, where the dark blue region belongs to the basin of the destination attractor. The basin structures are computed, as follows. In each panel, the center point, with position $\vec{c_0}$ in the phase space, is chosen to be within the basin of attraction and is thus blue. Different perturbations are then applied: $\vec{h} = \epsilon_1 \vec{h}_1 + \epsilon_2 \vec{h}_2$, where $\epsilon_1, \epsilon_2 \in [-10, 10]$ to the center points. Whether the reservoir memory stays in the destination attractor after the perturbation determines if the point at (ϵ_1, ϵ_2) (corresponding to the point $\vec{c_0} + \vec{h}$ in the high-dimensional phase space) is within the basin or not. It can be seen that, in terms of the relative sizes of the blue and orange regions as well as their structures, there is strong correlation between each panel in Fig. S8(A) and the corresponding three-panel column in Fig. S8(B). The structural correspondence is particularly remarkable. For example, the riddled structure in the third panel in the first row of Fig. S8(A) also appears in the third column of three panels in Fig. S8(B), and the "clean" boundary in the first panel in the second row of Fig. S8(A) can also be observed in the corresponding panels in Fig. S8(B).

The structural correlations suggest that the successful switching regions in Fig. S8(A) are the projections of the basin of attraction of the destination attractors. Furthermore, as the memorized attractors are confined in similar three-dimensional phase-space regions of their original dynamical systems, the corresponding regions that they reside in the high-dimensional phase space of the reservoir network should also be similar. However, the results in Fig. S8(B) indicate that the basins of attractions of different attractors can be quite different in terms of their sizes and structures. It is these differences that determine the success rate of memory switching.

Supplementary Note 8. PERFORMANCE OF OUR FEEDBACK CONTROL STRATEGY FOR HIGHER SWITCH SUCCESS RATES UNDER DIFFERENT PARAMETERS

In the main text, we present the performance of our feedback control strategy in Fig. 3 (E) with a moderate set of control parameters. The entire recall procedure under this control strategy is essentially a loop of trial and error. Within each iteration, there is a perturbation phase and a

classification phase. We apply random noises with a certain amplitude and temporal length to the memory RC in the perturbation phase, and then use the classifier RC to provide feedback information on whether the target state is reached. Figure S9 shows the performance of this strategy for indexed memory RC under different parameters. We vary the temporal length of random perturbation and the noise levels in each iteration of the trial and error loop. All the random perturbations are implemented by Gaussian white noise with standard deviation σ_p. The tests are run on the same ensemble of 25 memory RCs as in the main text, each trained with 6 attractors. Figure S9 (C2) is also the panel shown in the main text. It can be considered as the optimal one in all the 12 different combinations of strategy parameters. A moderate time length (around 10 steps) enables it to utilize almost the full potential of this method without taking too much time or computational resources. A moderate noise level (around $\sigma_p = 1$) also leads to a high overall correction success rate.

Supplementary Note 9. DYNAMICAL PROCESS OF RETRIEVAL IN INDEX-FREE RESERVOIR-COMPUTING MEMORY

We provide a further demonstration of the dynamic process of retrieval in index-free reservoir memory systems. As discussed in the main text, the goal of the process is to reach a target trajectory $g[u(t)]$ from a random initial state of the memory dynamical system, as illustrated in Fig. S12. In particular, in Fig. S12 (A), the four panels show four cases of random initial state (dashed red curves) approaching the target trajectory $g[u(t)]$ (solid purple curves) during the warming phase. The dashed red curves can approach the purple curves after one or two dozen steps, in agreement with the threshold values in Fig. 5(A) in the main text. Figure S12(B) shows how the warming data for the dashed red and solid purple curves in Fig. S12(A) are prepared. The echo state property of reservoir computing stipulates that the dynamical output trajectory can approach the target attractor after sufficient warming. The purple curves in Fig. S12(A) are simulated by the trajectories of the reservoir memory system after 400 steps of warming.

Supplementary Note 10. DEPENDENCY OF MEMORY RETRIEVAL IN INDEX-FREE RESERVOIR COMPUTERS WITH PARTIAL INFORMATION ON THE SPECIFIC MISSING DIMENSIONS

In the main text, we demonstrate how our index-free memory RC can still function and recall target states with partial cues missing some dimensions. However, we only show one missing-dimension scenario for each dimensionality in the main text, while there are more possible combinations of missing dimensions in the three-dimensional target states we test. It could be interesting to see how the retrieval performance depends on the specific combinations of dimensions that are missing. Here, we demonstrate comprehensive results on all the possibilities of missing dimensions with all the six attractors we test. The results are shown in Fig. S13 and Fig. S14. Comparing the six attractors, we observe that the thresholds of the cue length where the success rate begins to rise significantly larger than a random recalling are postponed similarly to the results shown in Fig. 6 in the main text. This again verifies that the threshold of cue length is a feature of the RC structure and properties rather than a feature of the specific target attractor, with the same way of rewiring feedback loops yielding the same thresholds. A decrease in the saturated success rate is

also observed in multiple cases, and is more frequent for the 1D cues than the 2D cues, as more information is lost. Among the four chaotic attractors, the Lorenz attractor (in panel (C)) can always reach a 100% success rate. Both the chaotic Rossler system and the chaotic food chain system suffer no decrease in the saturated success rate except in the sole case where only the third dimension is lost. The most intriguing case is with the periodic Sprott system, where missing the second dimension alone would result in a worse saturated success rate than missing two dimensions. This suggests that having more dimensions hidden during the retrieval does not always make the success rate worse; the relationship between missing dimensions and the change in success rate is much more complicated and system-dependent. Further research is necessary to unveil a possible generic understanding of these interesting phenomena.

Supplementary Note 11. EFFECTS OF NOISE AND RANDOM ITINERANCY

We apply independent Gaussian white noise of standard deviation of σ_n to each neuron in the reservoir network, for both index-based and index-free memory systems. For small noise, the output of the reservoir memory contains small random fluctuations. For large noise, the dynamics of the reservoir network are stochastic without any distinguishable dynamical patterns. For intermediate noise, an intermittent behavior between the memorized attractor and some random states arises for the index-based reservoir memory. For the index-free memory, because all the memorized attractors coexist in the phase space of the reservoir network, there is a random itinerary among the attractors. These results are exemplified in Figs. S15 and S16 for the index-based and index-free reservoir memory systems, respectively.

SUPPLEMENTARY FIGURES

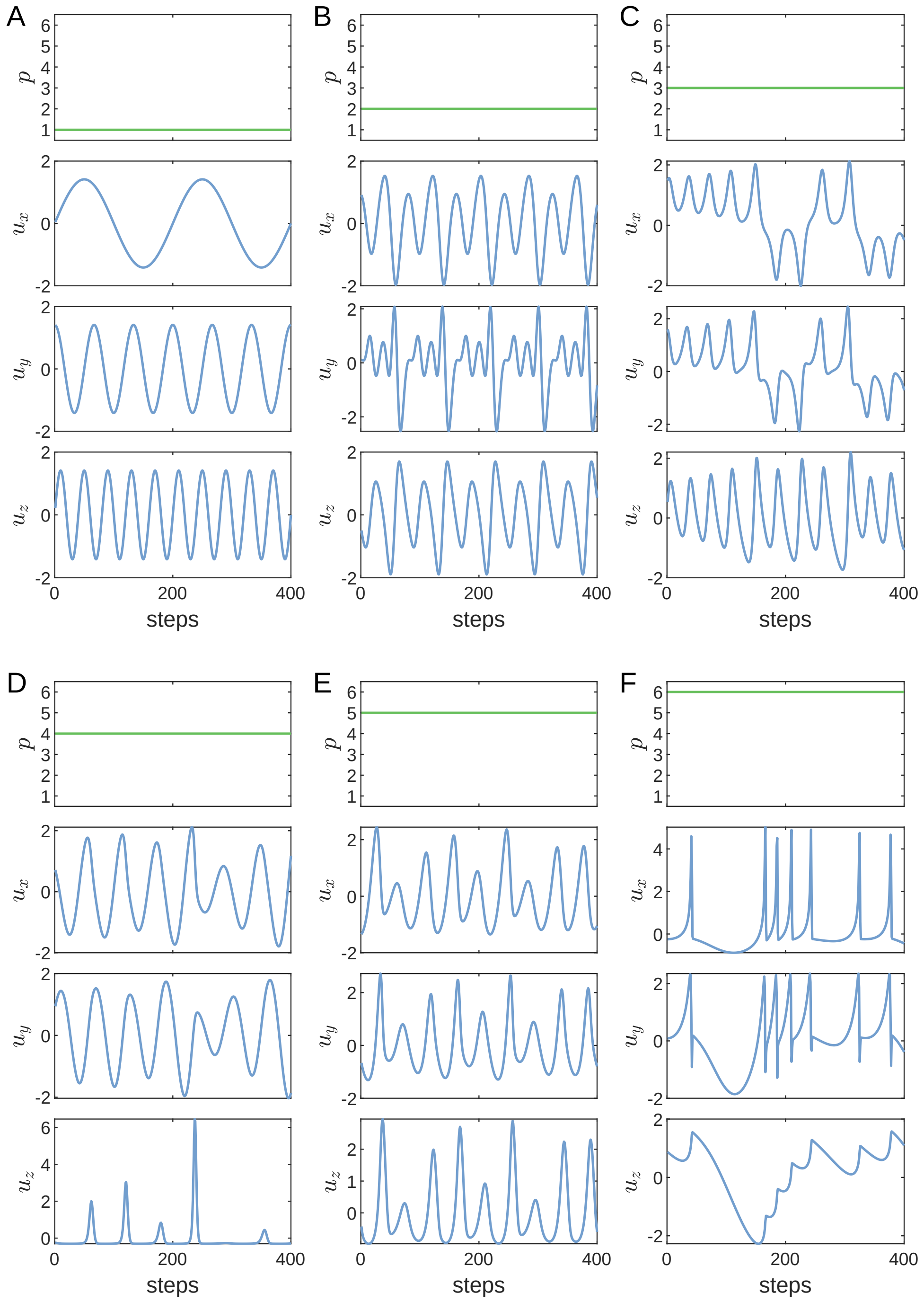

FIG. S1. Training data for the six attractors in Fig. 1(B) in the main text. The attractors are (A) a periodic Lissajous attractor, (B) a periodic attractor of the Sprott system, (C) the classic chaotic Lorenz attractor, (D) the classic chaotic Rössler attractor, (E) a chaotic attractor from food chain system, and (F) a chaotic attractor from the HR neuron system. The phase space for all six attractors is three-dimensional. During training of each attractor, a constant index value p is injected into the recurrent neural network through the index channel for index-based reservoir-computing memory, where no index channel is needed for index-free memory.

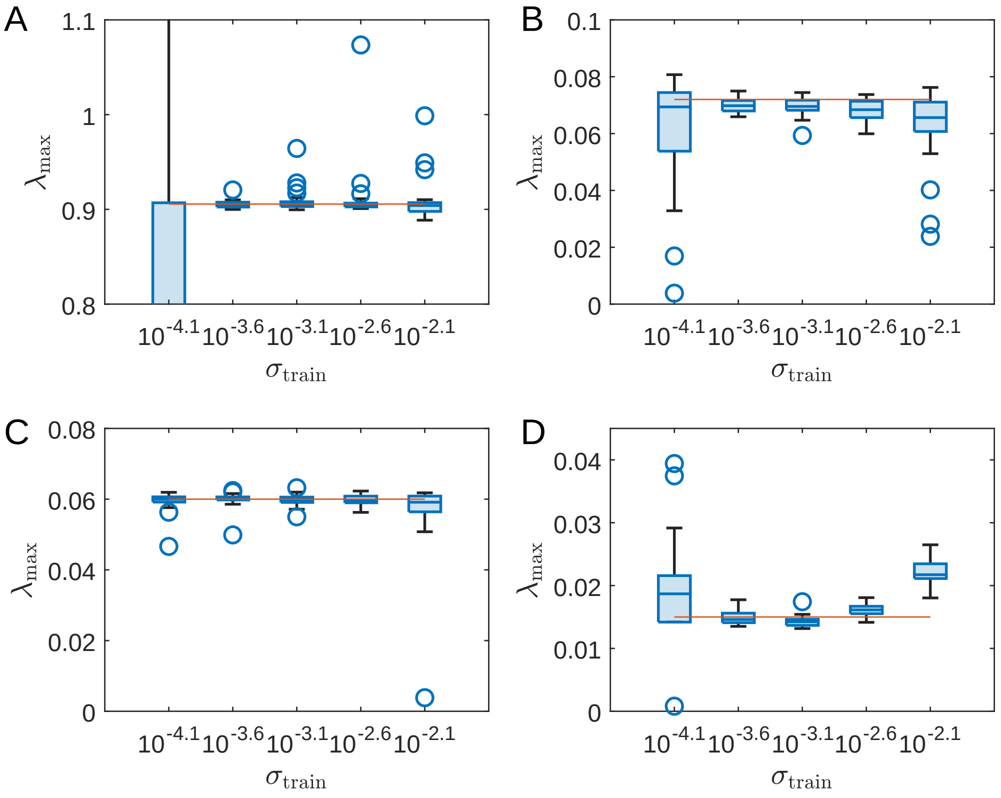

FIG. S2. Maximum Lyapunov exponent $\lambda_{\max}$ of the chaotic attractors reconstructed from the recalls of the memory RCs under different levels of training noise σ_{train}. The chaotic target states in each panel are (A) the Lorenz system, (B) the Rossler system, (C) the chaotic food chain system, and (D) the HR system. The red horizontal line represents the ground truth value of $\lambda_{\max}$ for each system. The upper and lower edges of the boxes represent the upper and lower quartiles of the resulting $\lambda_{\max}$ from 30 different memory RCs. The horizontal lines inside the boxes represent the median values of these resulting $\lambda_{\max}$. The outliers are shown by the circles, which are values that are more than 1.5 times of the interquartile range away from the top or bottom of the box. The upper whisker connects the upper quartile to the nonoutlier maximum (the maximum data value that is not an outlier), and the lower whisker connects the lower quartile to the nonoutlier minimum (the minimum data value that is not an outlier). The optimal noise level we have from the hyperparameter optimization is $\sigma_{\text{train}} = 10^{-3.1}$. It appears that when the noise level is around the optimal level, the majority of the $\lambda_{\max}$ values from the memory RCs agree with the ground truth values well. The index-based memory RCs tested here use the hyperparameter set Indexed #1 with $N = 1,200$. The training length for each target state is 6,000 steps. The maximum Lyapunov exponents $\lambda_{\max}$ are calculated from running the recalled state for 200,000 steps of iteration.

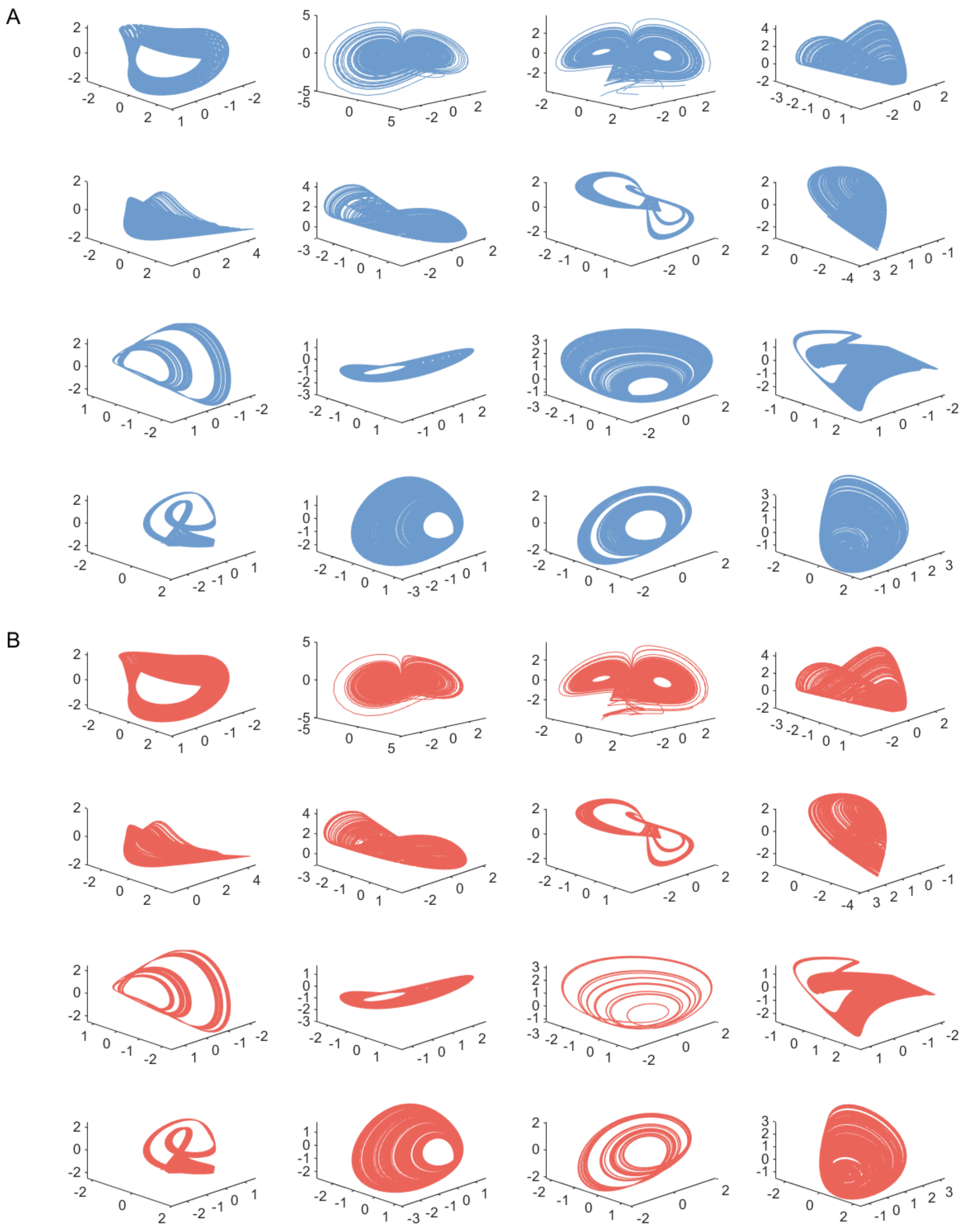

FIG. S3. Performance of index-based reservoir memory for memorizing 16 chaotic attractors. (A, B) Target (ground truth) and retrieved attractors, respectively. The 16 attractors are those in Fig. 1(C) in the main text. All the chaotic attractors can be successfully stored and faithfully recalled. Upon retrieval of any attractor, the reservoir system can generate an arbitrarily long trajectory on the attractor.

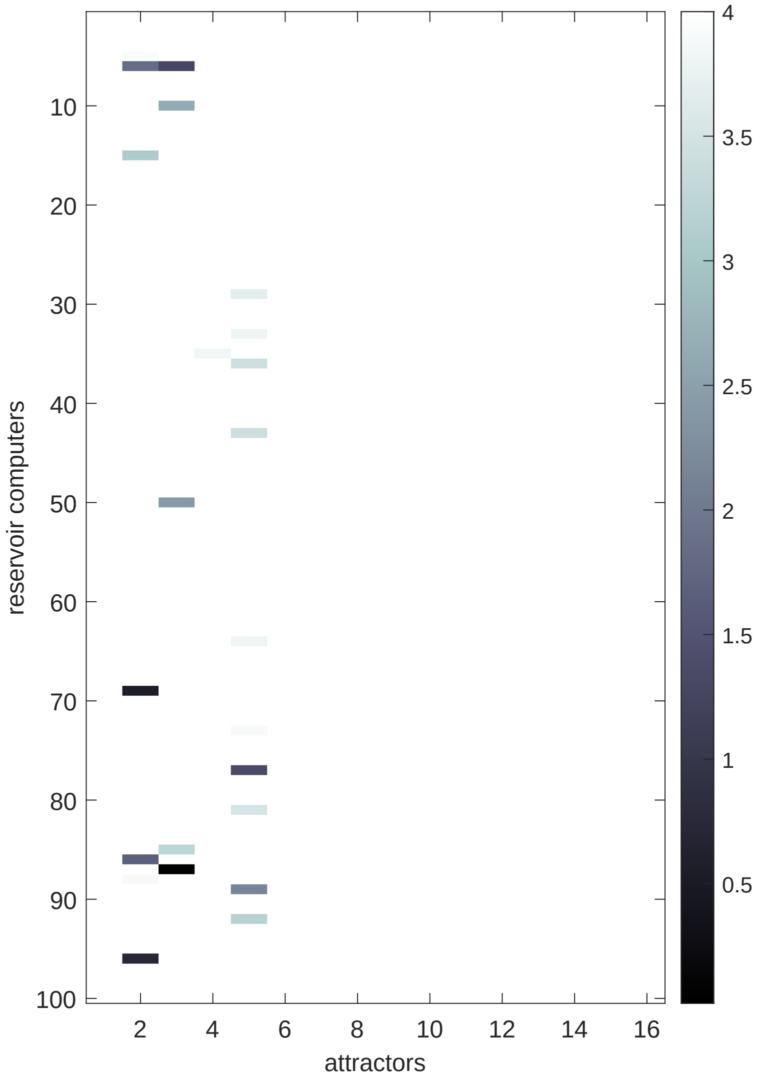

FIG. S4. Performance of index-based reservoir memory for memorizing 16 chaotic attractors, with a two-dimensional encoding where the order of the coding is randomized. The color represents the prediction horizon (by the unit of average period, which is the average temporal distance between two local maximums in the target state). We train and test 100 different reservoir computers. The 16 chaotic attractors are fixed (as the ones shown in Fig. S3), but which index each attractor is assigned is randomized. The prediction horizon is defined as the maximum temporal length during a recall testing that, in none of the dimensions of the target system, the deviation between the RC-generated trajectory and the ground truth target state is larger than 10% of the maximum value minus the minimum value in the target state. Our result suggests that our approach can successfully memorize and recall almost all the attractors regardless of how the index values are assigned.

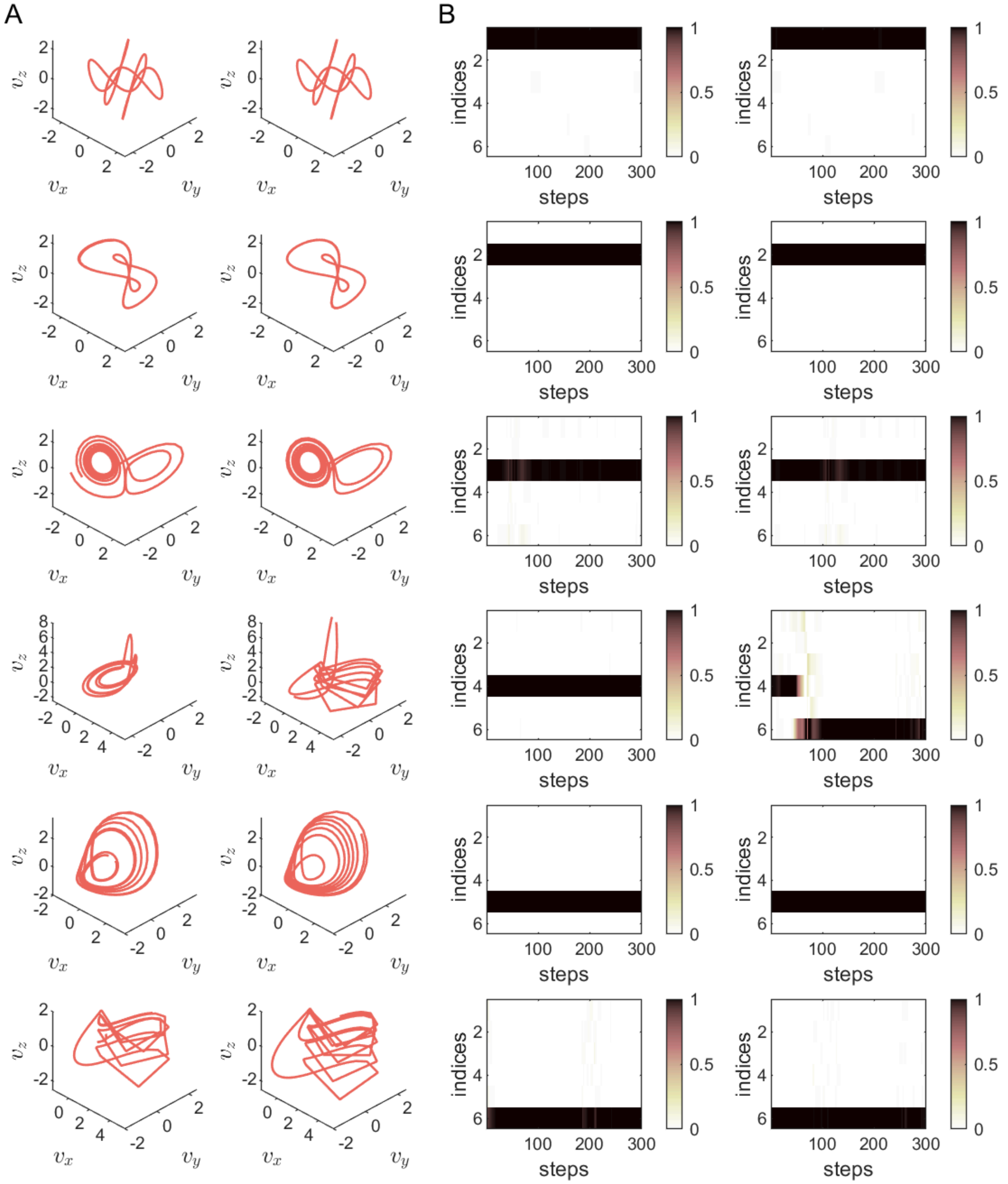

FIG. S5. Working example of the reservoir-computing-based classifier. (A, B) Exemplary input time series and output classifying results, respectively. The time series are generated by the reservoir memory system during the retrieval process. A dark stripe in the correct row of the classifier output indicates that the time series tested is from the correct memorized attractor. An example of failed retrieval is shown in the fourth row where, after recalling the correct Rössler attractor for a short period of transient time, the system switches to the sixth attractor, as shown in (A). In the corresponding panel in (B), the dark stripe in the output at index $p = 4$ breaks and a new stripe at index $p = 6$ is formed.

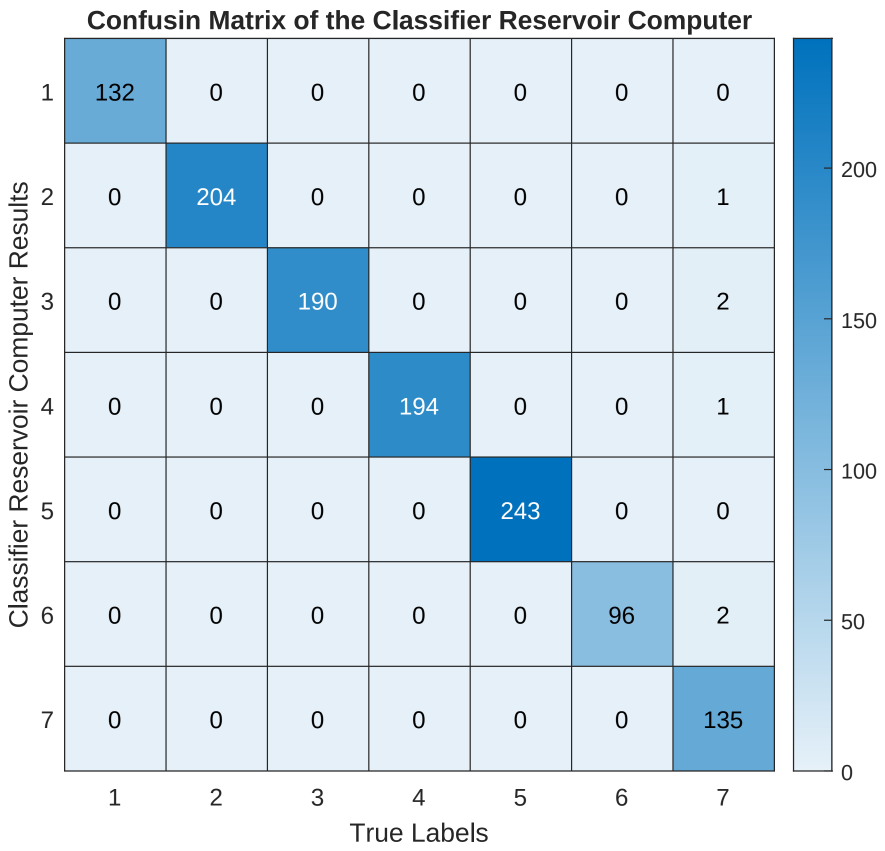

FIG. S6. Confusion matrix of the reservoir-computing-based classifier. Labels 1 to 6 represent the six target states, while label 7 represents an untrained state in a failed recall. The RC classifiers show high accuracy in classifying different target states as well as distinguishing untrained states. Among all the 1200 trials among 8 different memory RCs, there are only 6 trials where the classifier RC results are different from the human labeler. All these 6 trials are caused by the rather ambiguous and hard-to-define boundary between a successfully recalled target state and a failed one.

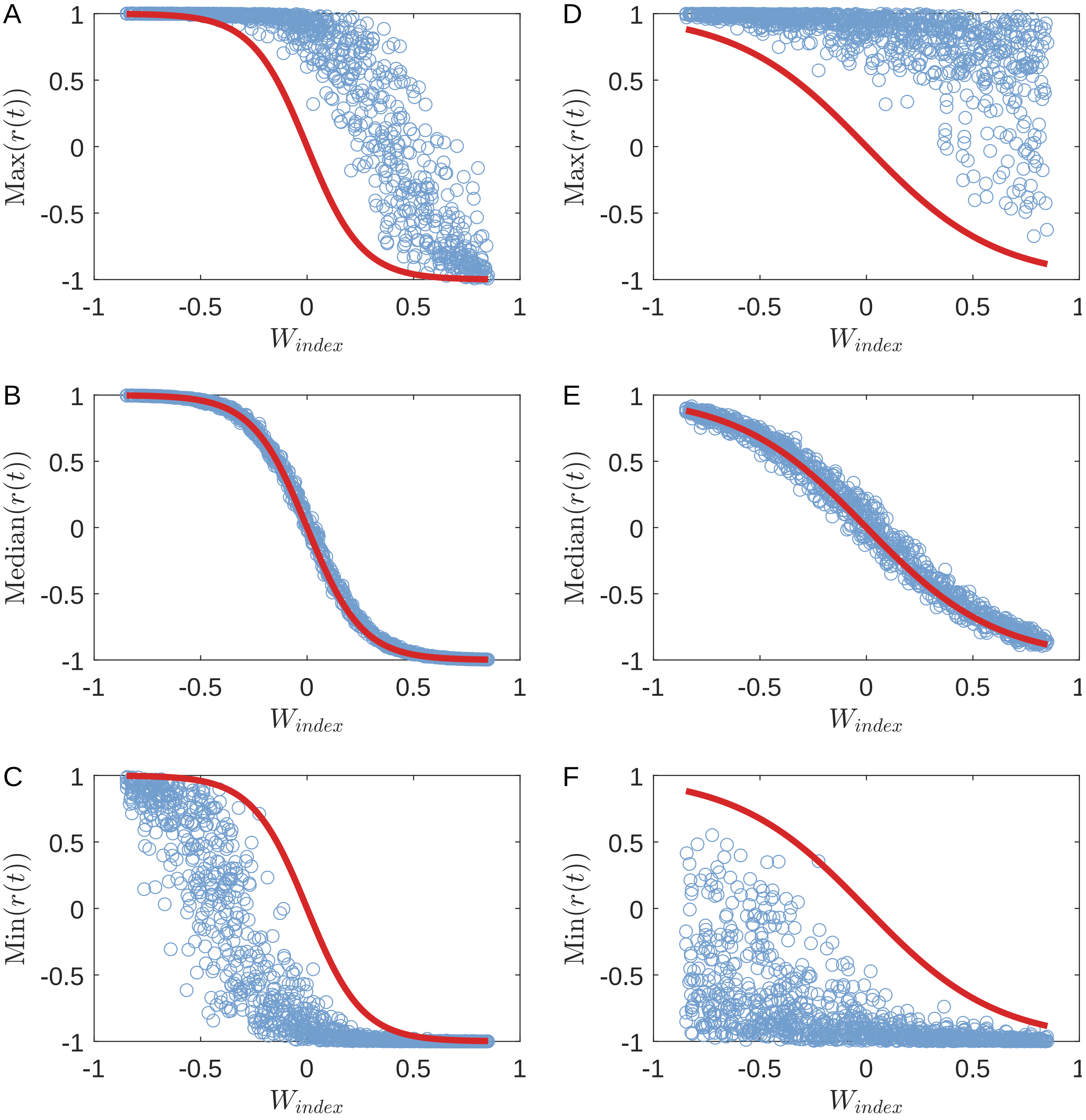

FIG. S7. Effects of index values on the functional regions of artificial neurons in the reservoir network. (A, D) The maximum (B, E) median, and (C, F) minimum values of each neuron for two different target attractors in an index-based reservoir memory trained to store the six attractors displayed in Fig. 1(B) in the main text, where the left and right columns are for attractors 1 and 3, respectively. Each blue circle represents the state of a neuron in the reservoir network, the horizontal coordinate of which is the value of the entry in W_{index} connected to that neuron. The red curves represent the function $\tanh(W_{\mathrm{index}} p_i)$, which fits well the median values of the oscillating neurons [(B) and (E)]. The results demonstrate how the oscillatory patterns of the neurons in the index-based reservoir memory are tuned by the index value through W_{index}.

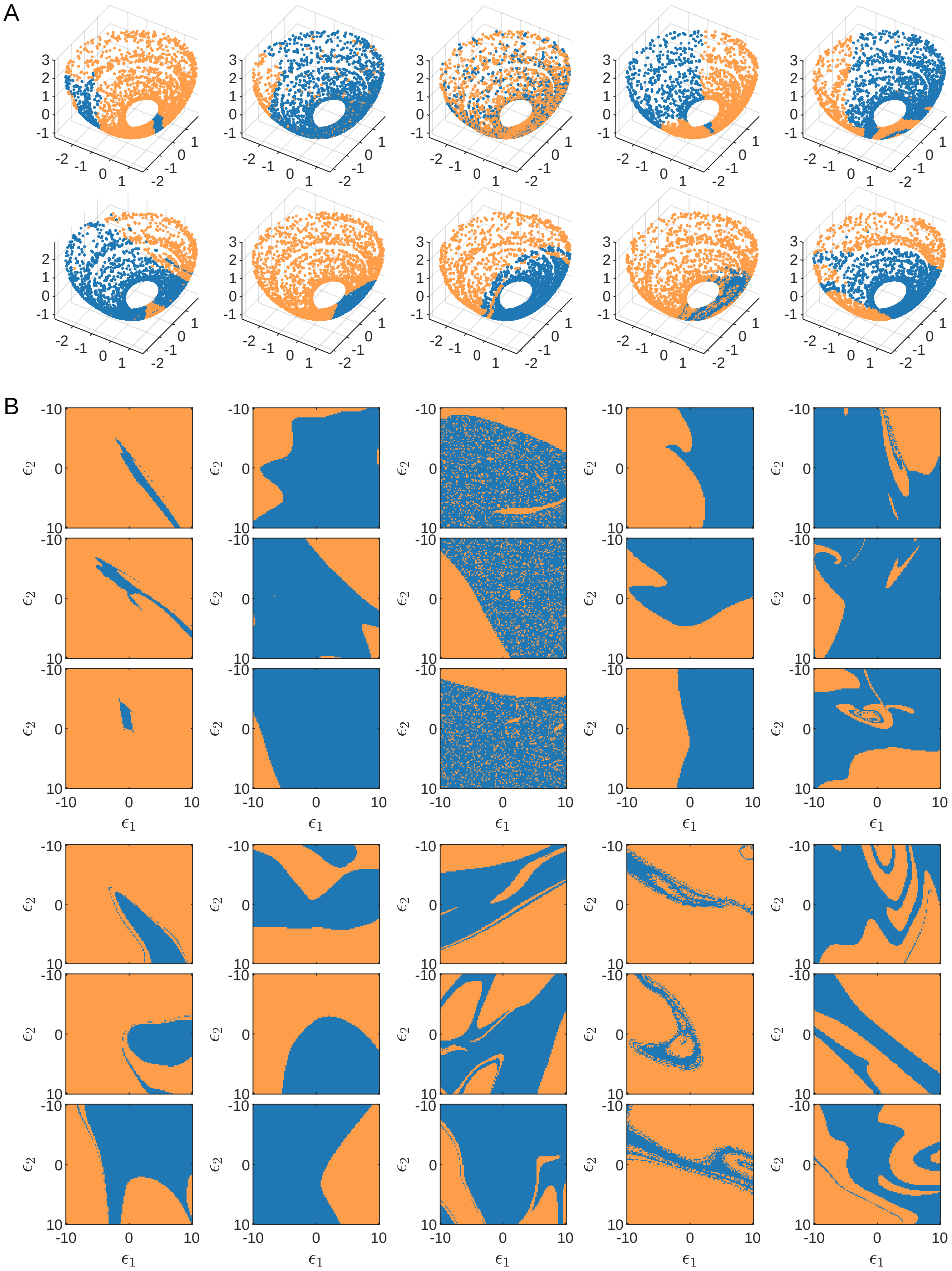

FIG. S8. Switching success/failure landscape and basin structures in index-based reservoir computer with multiple memory states. (A) Regions of successful and failed switching from the same starting attractor (No. 11 in Fig. 3(C) in the main text) to ten different destination attractors, where each dot is the point at which the switching begins. The darker blue and orange dots correspond to successful and failed switchings, respectively. (B) The corresponding basin structures of the ten different destination attractors in the high-dimensional phase space of the reservoir network from the same starting attractor in (A), where each panel in (A) corresponds to a column of three different 2D slices (panels) in (b), in the same order. In each panel, the darker blue regions denote the attracting basin of the corresponding destination attractor, while the orange regions do not belong to the basin of attraction and lead to failed switching as the reservoir output can be some irrelevant dynamical states (e.g., a fixed point).

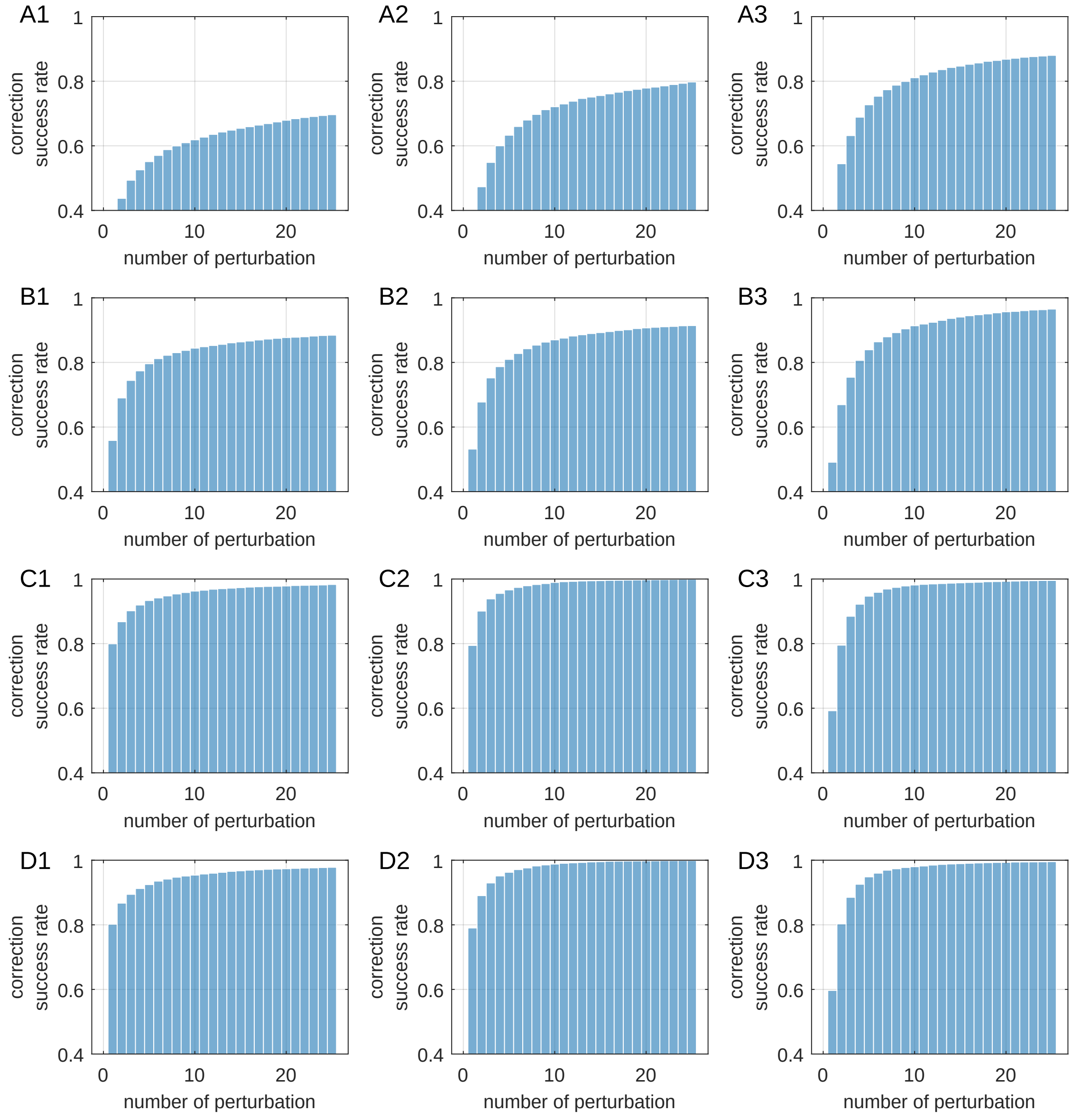

FIG. S9. Performance of our second control strategy (with a classifier RC and random perturbations) under different setting parameters. The length of a single run of random perturbation is (A1, A2, A3) 1 step, (B1, B2, B3) 3 steps, (C1, C2, C3) 10 steps, and (D1, D2, D3) 30 steps. The noise levels are (A1, B1, C1, D1) $\sigma_p = 0.3$, (A2, B2, C2, D2) $\sigma_p = 1$, and (A3, B3, C3, D3) $\sigma_p = 3$. We observe that there is little difference between the (C1, C2, C3) row and the (D1, D2, D3) row, while the (B1, B2, B3) and (A1, A2, A3) rows have significantly worse performance. This result suggests that one needs the perturbation length to be not way too short, but does not need it to be very long either as it will not enhance the performance much. The comparisons among the three columns also suggest the existence of an optimal moderate noise level.

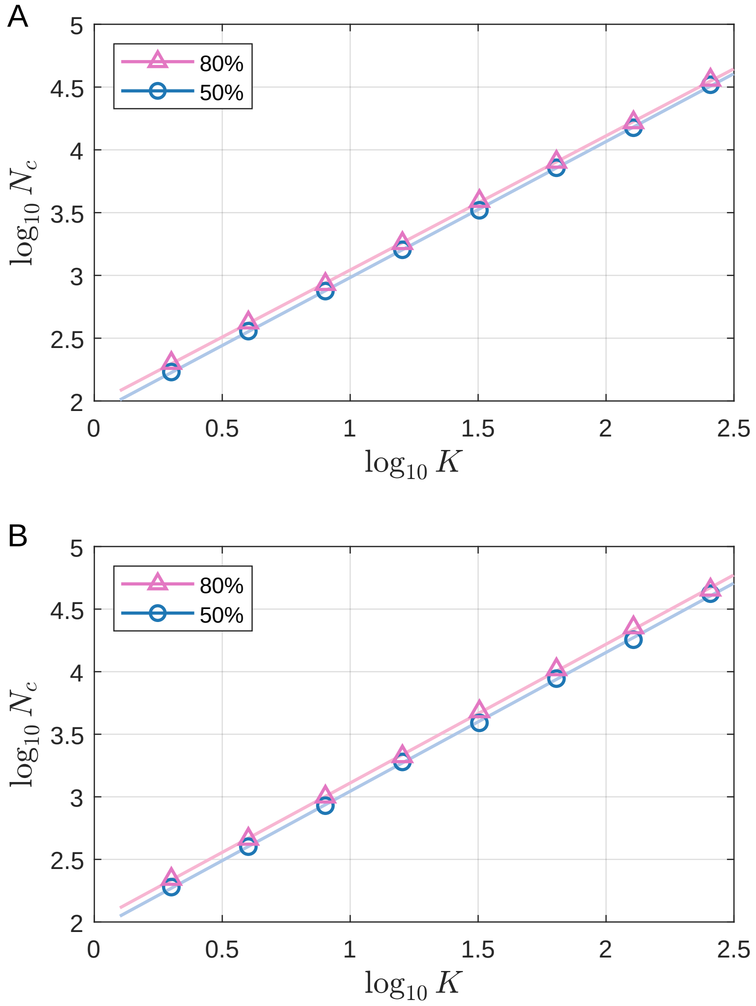

FIG. S10. Comparisons between the scaling laws plotted by the 50% success rate versus the 80% success rate. Shown are two pairs of examples with indexed memory RC with the on-hot coding on Dataset #1 with (A) the region-based performance measure and (B) the prediction-horizon-based performance measure. The scaling laws do not appear to differ with different success rates, except for a constant factor.

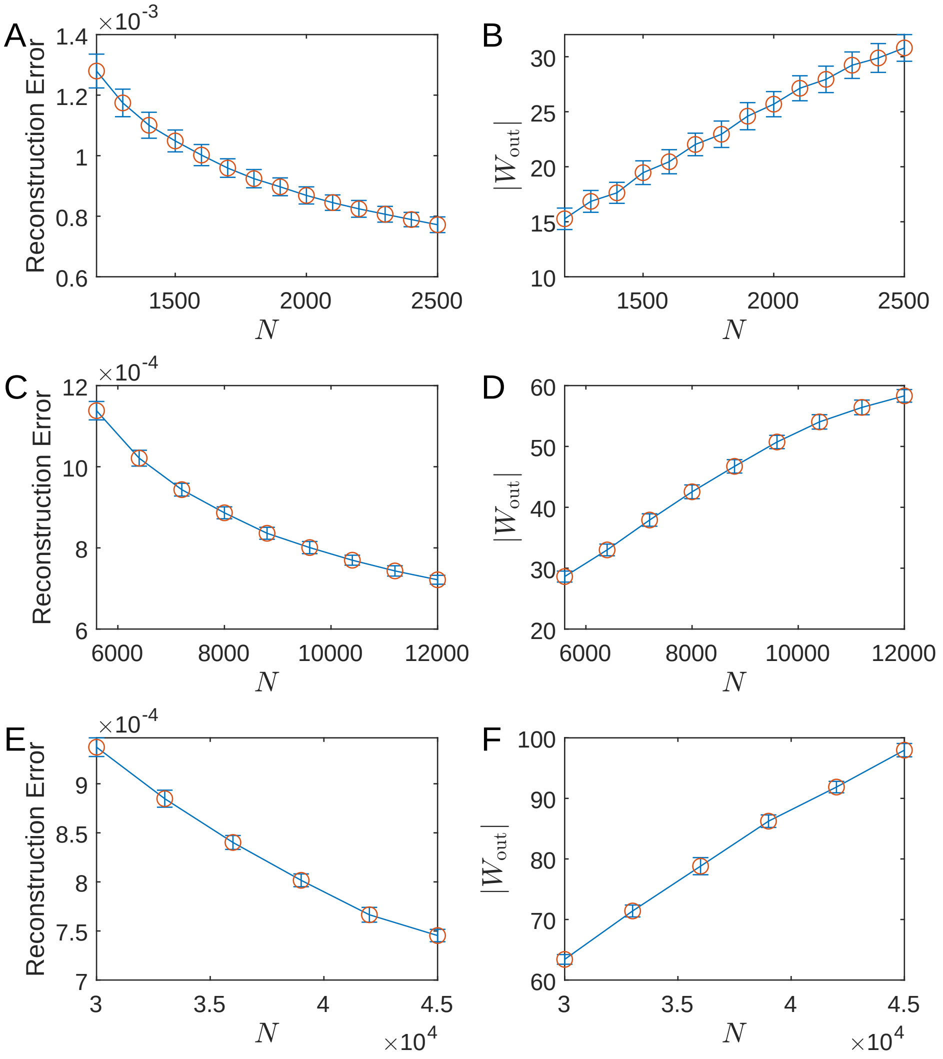

FIG. S11. Reconstruction error (A, C, E) and 2-norm of the $W_{\rm out}$ (B, D, F) of the index-based memory RC near the critical network size N_c. All the memory RCs in this figure are trained on Dataset #1 with a one-hot coding. Panels (A, B) are from memory RCs trained with $K = 16$ attractors, with $N_c = 1,600$ with the region-based measure and $N_c = 1,900$ with the prediction-horizon-based measure. The results are averaged over 200 random RCs, and the error bar represents the standard deviation within this ensemble. Panels (C, D) are from memory RCs trained with $K = 64$ attractors, with $N_c = 7,200$ with the region-based measure and $N_c = 8,800$ with the prediction-horizon-based measure. The results are averaged over 120 random RCs, and the error bar represents the standard deviation within this ensemble. Panels (E, F) are from memory RCs trained with $K = 256$ attractors, with $N_c = 33,000$ with the region-based measure and $N_c = 42,000$ with the prediction-horizon-based measure. Here, the reconstruction error is calculated by the RMSE on the training data of all target states after training. The results are averaged over 25 random RCs, and the error bar represents the standard deviation within this ensemble. As the number of memory states K increases for more than an order of magnitude, the reconstruction error around N_c is always around 8×10^{-4} to 1×10^{-3}. The 2-norm of the $W_{\rm out}$ around N_c is increasing, but not as fast as K.

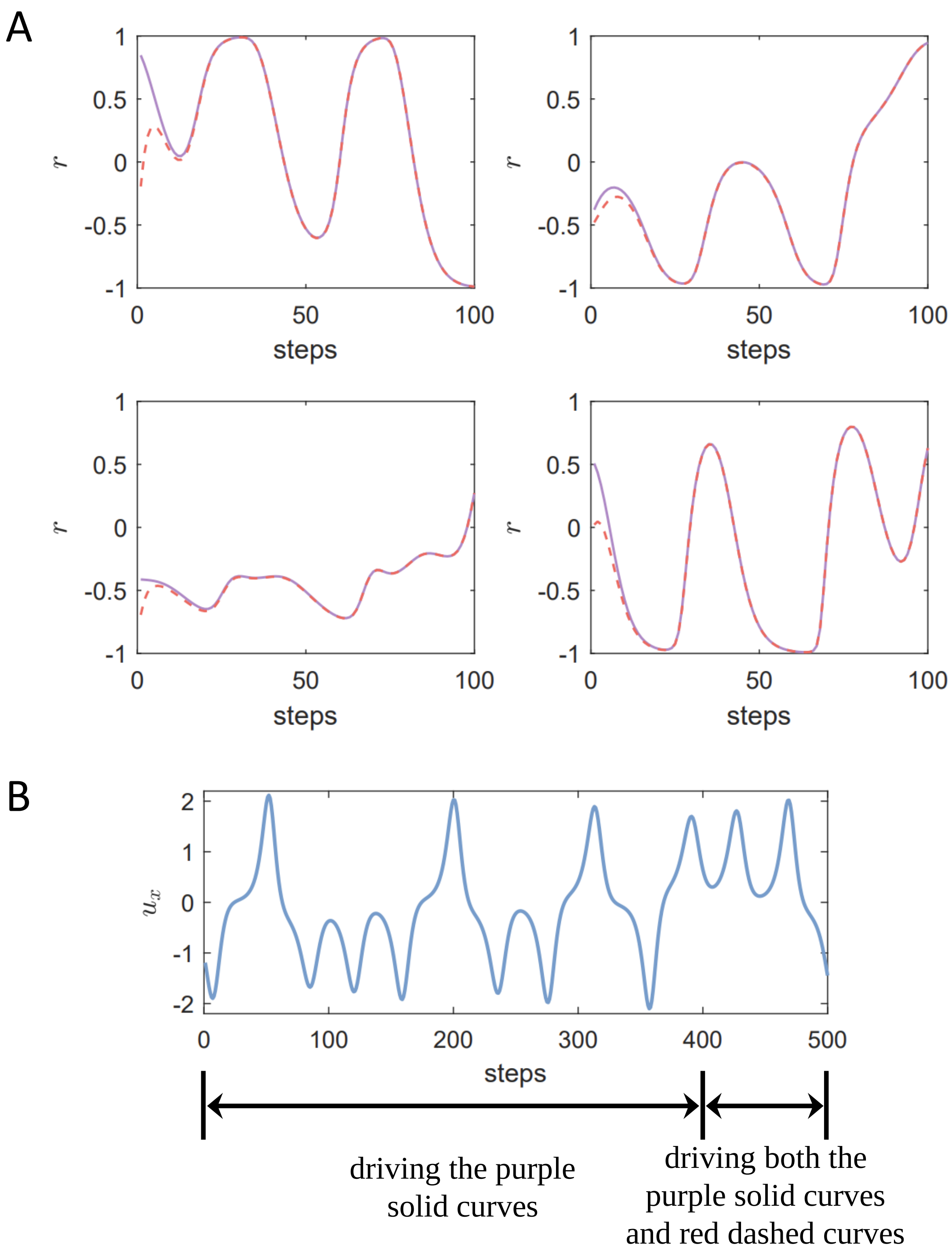

FIG. S12. Dynamical process of retrieval in index-free reservoir-computing memory. Presented is a demonstration of how the network state of index-free reservoir memory approaches the target trajectories. (A) Four random examples of the network dynamical state (dashed red curves) approaching the target trajectories (solid purple curves) after one or two dozen steps. (B) Warming data for the dashed red and solid purple curves in (A).

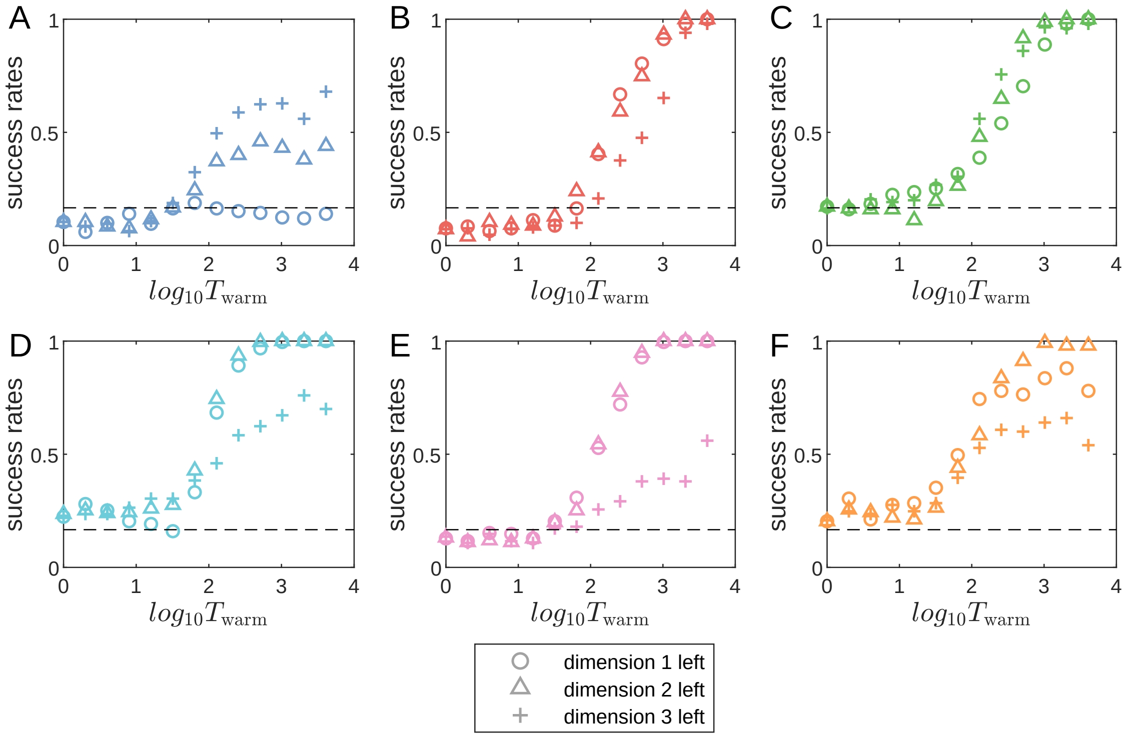

FIG. S13. Attractor retrieval with partial cues in index-free memory RCs for all possible scenarios with only one dimension left in the originally three-dimensional cues. (A-F) Success rate of retrieval versus the cue length for the six attractors in Fig. 1B in the main text (from left to right)). All values of the hyperparameters, training settings, and target memory states are the same as those in Fig. 6 in the main text.

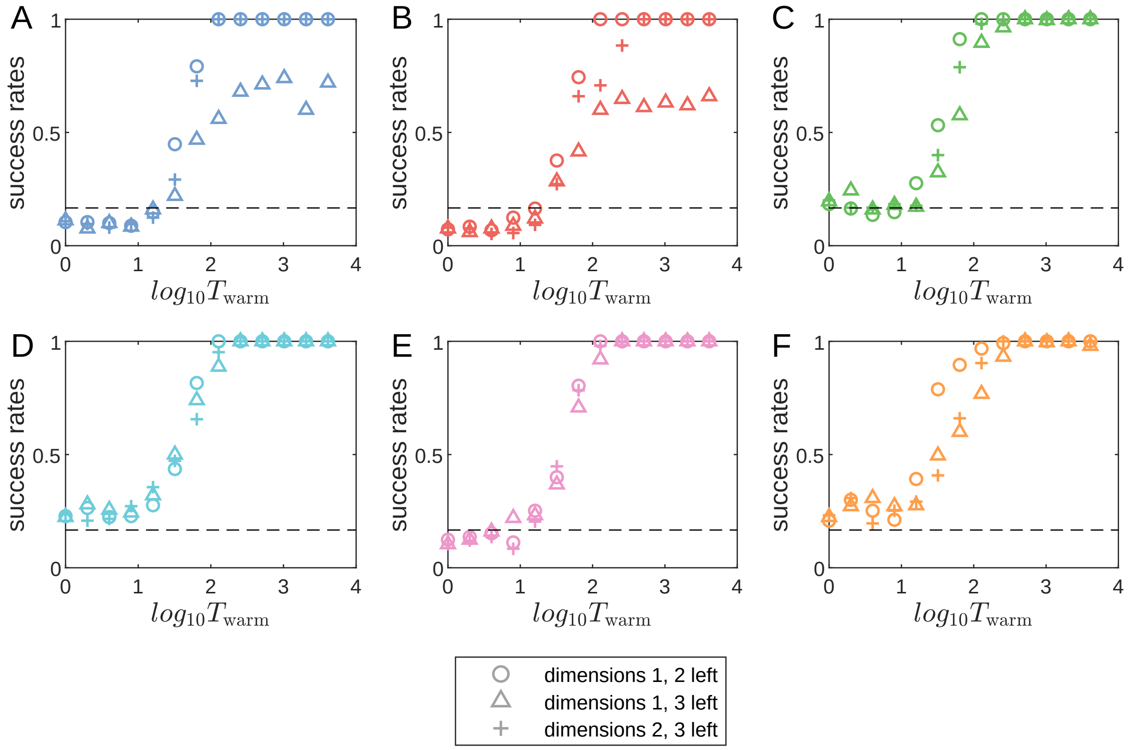

FIG. S14. Attractor retrieval with partial cues in index-free memory RCs for all possible scenarios with two dimensions left in the originally three-dimensional cues. (A-F) Success rate of retrieval versus the cue length for the six attractors in Fig. 1B in the main text (from left to right)). All values of the hyperparameters, training settings, and target memory states are the same as those in Fig. 6 in the main text.

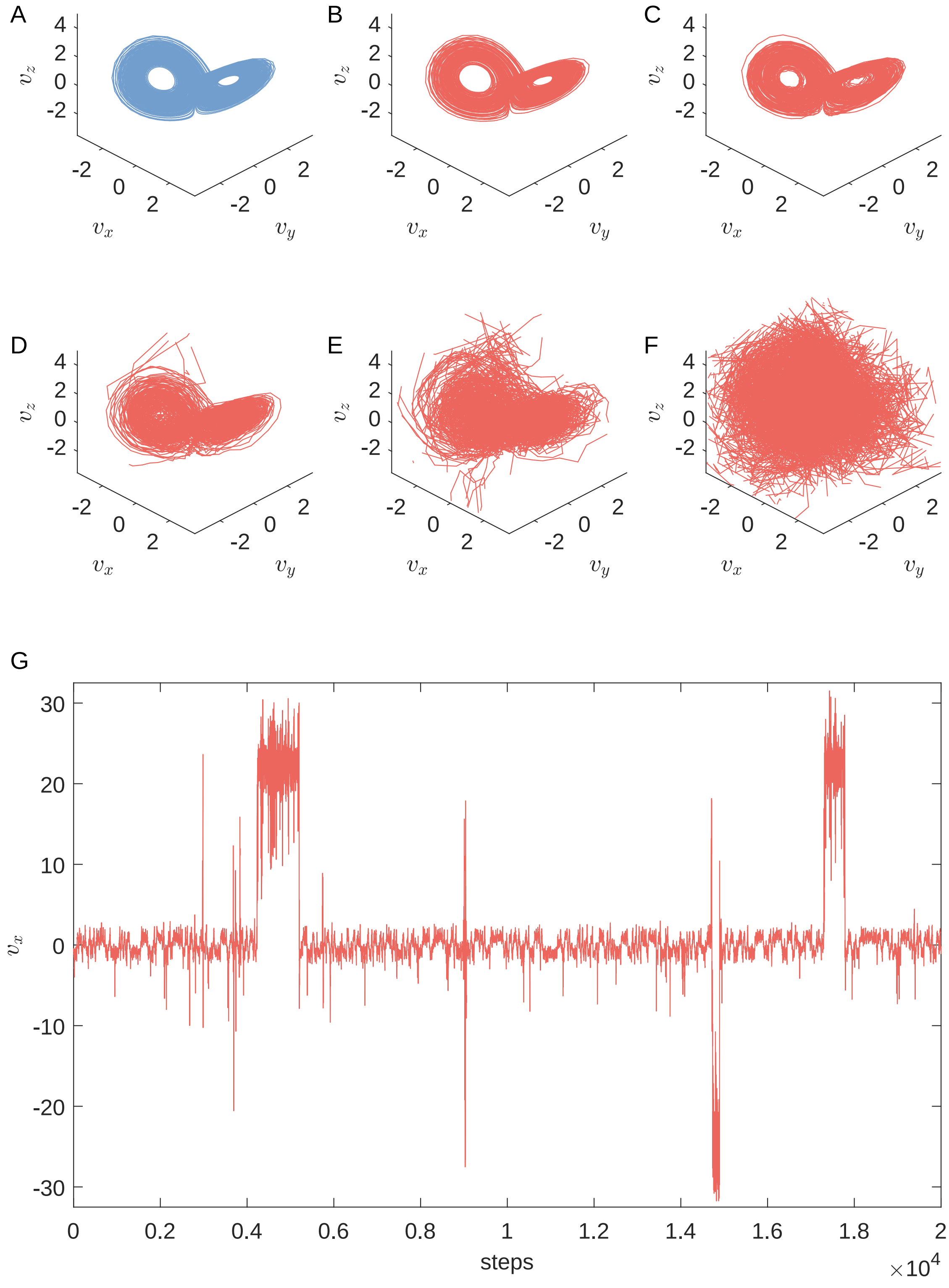

FIG. S15. Effects of noise in index-based reservoir memory system. (A) Original attractor (ground truth). (B-F) Recalled attractors under different levels of noise applied to each neuron in the reservoir network: (B) $\sigma_n = 10^{-4}$, (C) $\sigma_n = 10^{-3.5}$, (D) $\sigma_n = 10^{-3}$, (E) $\sigma_n = 10^{-2.5}$, and (F) $\sigma_n = 10^{-2}$. (G) Intermittency between the memorized attractor and some random untrained states for $\sigma_n = 10^{-2.5}$. When the output v_x is within the interval $(-3, 3)$, the output trajectory is close to the true memorized attractor.

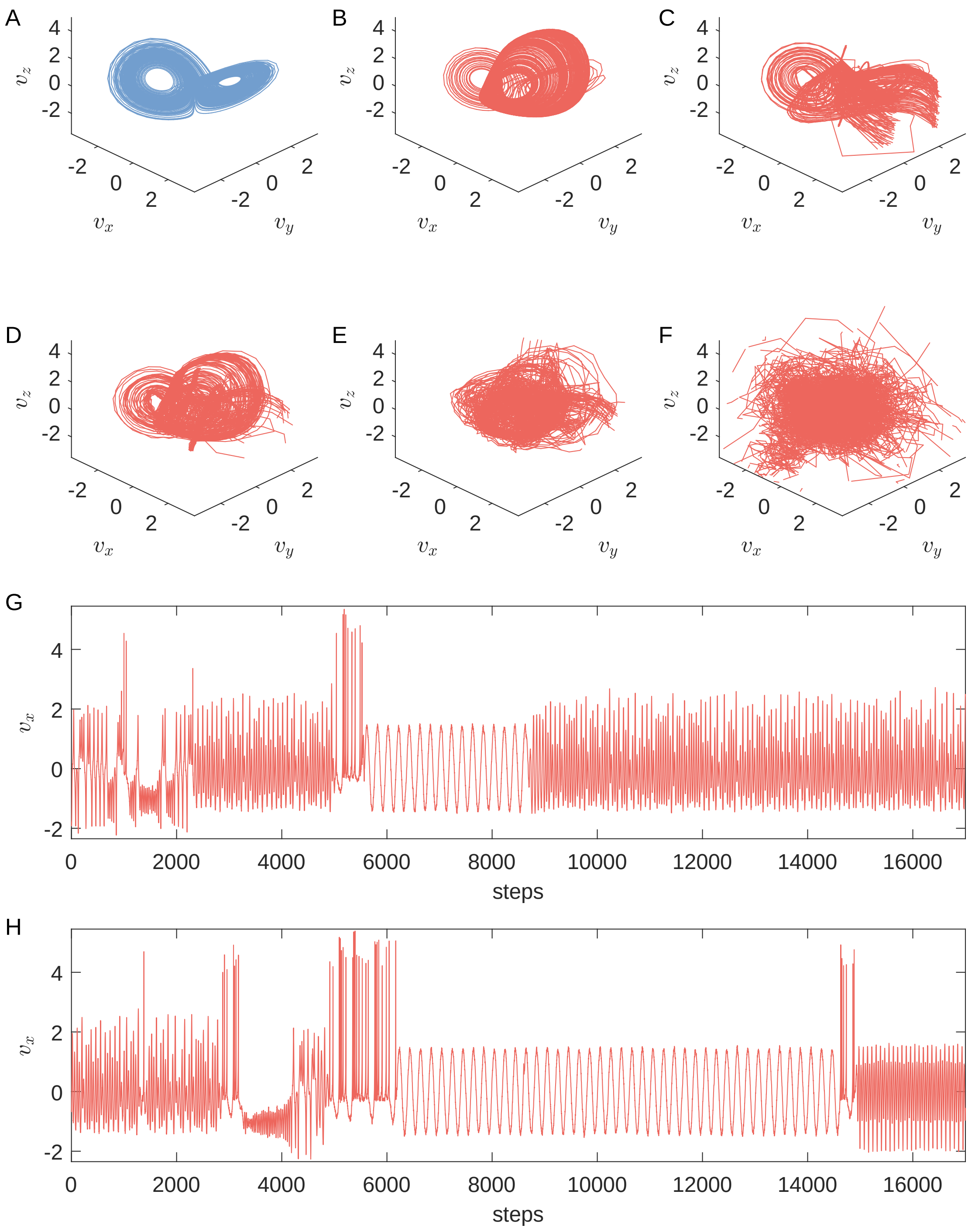

FIG. S16. Effects of noise in index-free reservoir memory. (A) Original attractor (ground truth). (B-F) Recalled attractors under different levels of noise: (B) $\sigma_n = 10^{-4.5}$, (C) $\sigma_n = 10^{-4}$, (D) $\sigma_n = 10^{-3.5}$, (E) $\sigma_n = 10^{-3}$, and (F) $\sigma_n = 10^{-2.5}$. (G-H) Two examples of random itinerary among the memorized attractors for $\sigma_n = 10^{-3.5}$. In (G), the itinerary order is: chaotic Lorenz attractor → chaotic food-chain attractor → chaotic HR neuron attractor → Lissajous attractor → chaotic food-chain attractor. In (H), the itinerary is: chaotic food-chain attractor → HR neuron attractor → chaotic Lorenz attractor → chaotic Rössler attractor → chaotic HR neuron attractor → Lissajous attractor → chaotic HR neuron attractor → a periodic attractor.

SUPPLEMENTARY REFERENCES

[1] Kong, L.-W. Codes. GitHub: https://github.com/lw-kong/Long-Term-Memory-in-RC (2024).

[2] Zhai, Z.-M., Kong, L.-W. & Lai, Y.-C. Emergence of a resonance in machine learning. *Phys. Rev. Res.* **5**, 033127 (2023).

[3] Kong, L.-W., Fan, H.-W., Grebogi, C. & Lai, Y.-C. Machine learning prediction of critical transition and system collapse. *Phys. Rev. Res.* **3**, 013090 (2021).

[4] Fan, H., Kong, L.-W., Lai, Y.-C. & Wang, X. Anticipating synchronization with machine learning. *Phys. Rev. Resesearch* **3**, 023237 (2021).

[5] Kong, L.-W., Fan, H., Grebogi, C. & Lai, Y.-C. Emergence of transient chaos and intermittency in machine learning. *J. Phys. Complexity* **2**, 035014 (2021).

[6] Lu, Z. *et al.* Reservoir observers: Model-free inference of unmeasured variables in chaotic systems. *Chaos* **27**, 041102 (2017).

[7] Flynn, A., Herteux, J., Tsachouridis, V. A., Räth, C. & Amann, A. Symmetry kills the square in a multifunctional reservoir computer. *Chaos: An Interdisciplinary Journal of Nonlinear Science* **31**, 073122 (2021).

[8] Kong, L.-W. Data and codes. GitHub: https://github.com/lw-kong/Long-Term-Memory-in-RC (2023).

[9] Sprott, J. C. Some simple chaotic flows. *Phys. Rev. E* **50**, R647–R650 (1994).

[10] Blasius, B., Huppert, A. & Stone, L. Complex dynamics and phase synchronization in spatially extended ecological systems. *Nature* **399**, 354–359 (1999).

[11] Hindmarsh, J. L. & Rose, R. M. A model of neuronal bursting using three coupled first order differential equations. *Proc. R. Soc. Lon. Ser. B Biol. Sci.* **221**, 87–102 (1984).
